# Phylogeographic Pattern and Extensive Mitochondrial DNA Divergence Disclose a Species Complex within the Chagas Disease Vector *Triatoma dimidiata*

**DOI:** 10.1371/journal.pone.0070974

**Published:** 2013-08-05

**Authors:** Fernando A. Monteiro, Tatiana Peretolchina, Cristiano Lazoski, Kecia Harris, Ellen M. Dotson, Fernando Abad-Franch, Elsa Tamayo, Pamela M. Pennington, Carlota Monroy, Celia Cordon-Rosales, Paz Maria Salazar-Schettino, Andrés Gómez-Palacio, Mario J. Grijalva, Charles B. Beard, Paula L. Marcet

**Affiliations:** 1 Laboratório de Epidemiologia e Sistemática Molecular, Instituto Oswaldo Cruz – Fiocruz, Rio de Janeiro, Brazil; 2 Laboratory of Molecular Systematics, Limnological Institute of the Siberian Branch of the Russian Academy of Sciences, Irkutsk, Russia; 3 Instituto de Biologia, Universidade Federal do Rio de Janeiro, Rio de Janeiro, Brazil; 4 Entomology Branch, Division of Parasitic Diseases and Malaria, Centers for Disease Control and Prevention, Atlanta, Georgia, United States of America; 5 Instituto Leonidas e Maria Deane – Fiocruz Amazonia, Manaus, Brazil; 6 Área de Entomología Médica, Centro Universitario del Sur, Universidad de Guadalajara, Ciudad Guzmán, México; 7 Center for Health Studies, Universidad del Valle de Guatemala, Guatemala City, Guatemala; 8 Laboratory of Applied Entomology and Parasitology, Facultad de Ciencias Químicas y Farmacia, Universidad de San Carlos de Guatemala, Ciudad de Guatemala, Guatemala; 9 Laboratorio de Biología de Parásitos, Departamento de Microbiología y Parasitología, Universidad Nacional Autónoma de México, Ciudad de México, México; 10 Grupo de Biología y Control de Enfermedades Infecciosas, Instituto de Biología, Universidad de Antioquia, Medellín, Colombia; 11 Tropical Disease Institute, Department of Biomedical Sciences, Heritage College of Osteopathic Medicine, Ohio University, Athens, Ohio, United States of America; 12 Division of Vector-Borne Infectious Diseases, Centers for Disease Control and Prevention, Fort Collins, Colorado, United States of America; Universidade Federal do Rio de Janeiro, Brazil

## Abstract

**Background:**

*Triatoma dimidiata* is among the main vectors of Chagas disease in Latin America. However, and despite important advances, there is no consensus about the taxonomic status of phenotypically divergent *T. dimidiata* populations, which in most recent papers are regarded as subspecies.

**Methodology and Findings:**

A total of 126 cyt b sequences (621 bp long) were produced for specimens from across the species range. Forty-seven selected specimens representing the main cyt b clades observed (after a preliminary phylogenetic analysis) were also sequenced for an ND4 fragment (554 bp long) and concatenated with their respective cyt b sequences to produce a combined data set totalling 1175 bp/individual. Bayesian and Maximum-Likelihood phylogenetic analyses of both data sets (cyt b, and cyt b+ND4) disclosed four strongly divergent (all pairwise Kimura 2-parameter distances >0.08), monophyletic groups: Group I occurs from Southern Mexico through Central America into Colombia, with Ecuadorian specimens resembling Nicaraguan material; Group II includes samples from Western-Southwestern Mexico; Group III comprises specimens from the Yucatán peninsula; and Group IV consists of sylvatic samples from Belize. The closely-related, yet formally recognized species *T. hegneri* from the island of Cozumel falls within the divergence range of the *T. dimidiata* populations studied.

**Conclusions:**

We propose that Groups I–IV, as well as *T. hegneri*, should be regarded as separate species. In the Petén of Guatemala, representatives of Groups I, II, and III occur in sympatry; the absence of haplotypes with intermediate genetic distances, as shown by multimodal mismatch distribution plots, clearly indicates that reproductive barriers actively promote within-group cohesion. Some sylvatic specimens from Belize belong to a different species – likely the basal lineage of the *T. dimidiata* complex, originated ∼8.25 Mya. The evidence presented here strongly supports the proposition that *T. dimidiata* is a complex of five cryptic species (Groups I–IV plus *T. hegneri*) that play different roles as vectors of Chagas disease in the region.

## Introduction

Chagas disease (American Trypanosomiasis) is one of the most important parasitic diseases in Latin America with about 8–10 million people infected, 10–12 thousand deaths per year and ∼25 million at risk of infection [1], [2]. Humans acquire the disease when they come into contact with *Trypanosoma cruzi*-infected faeces of blood-sucking bugs of the subfamily Triatominae (Hemiptera: Reduviidae). As the most effective mechanism to prevent Chagas disease transmission relies on vector control strategies, substantial effort has been devoted to the study of the ecology, population structure and evolution of triatomine bugs (for review see [3]). *Triatoma dimidiata*, *T. infestans*, and *Rhodnius prolixus* are the main vectors of Chagas disease. Vector control programs have achieved remarkable success towards the elimination of *R. prolixus* and *T. infestans* in several regions of Central and South America, respectively [4]; *T. dimidiata* is currently the primary target of control efforts across its range [5], which spans from southern Mexico through Central America into Colombia, Peru, and Ecuador [6].

*T. dimidiata* occupies a wide variety of domestic and peridomestic environments, in both rural areas [7], [8] and urban settings [9], [10]. In the wild, it is also very versatile, and colonies have been found in a wide variety of ecotopes (e.g., rocky outcrops, trees, caves, and palm trees [11], [12], [13]).

Throughout its geographic distribution, *T. dimidiata* exhibits high phenotypic variability, which has caused considerable taxonomic controversy since the species description in 1811. A number of chromatic, morphometric, and antennal phenotype variants have been recognized; while some of them were regarded as geographic populations, others were formally described as subspecies or species (reviewed by [14]).

The first genetic evidence suggesting the existence of undescribed cryptic species within the *T. dimidiata* taxon was reported in 2001 [15]. Based on nucleotide sequence analyses of the ribosomal DNA second internal transcribed spacer (ITS-2), substantial differences were observed between one population from Yucatán (Mexico) and those from other localities in Mexico and Central America. Chromosome C-banding patterns, genome size [16], and mitochondrial cyt b sequence analyses [17] later corroborated these findings and extended the distribution of this new species into the forests of Petén, Guatemala. Bargues et al. [18] have proposed that all other *T. dimidiata* populations (including the closely related species *T. hegneri*) although also genetically distinct, but with distance values markedly lower than those for the particular population from Yucatán, should be regarded as subspecies. Genetic groups based on the subspecific criteria adopted by Usinger [19] were proposed: Group 1A (specimens from Chiapas in southern Mexico, Guatemala, Honduras, El Salvador, Nicaragua, Costa Rica, and Ecuador), which would correspond to *T. dimidiata dimidiata*; Group 1B (specimens from Panama and Colombia), corresponding to *T. dimidiata capitata*; and Group 2 (samples from central and northwestern Mexico, Guatemala and Belize), corresponding to *T. dimidiata maculipennis*. Results based on cuticular hydrocarbon patterns support the existence of these “three subspecies” and suggest the existence of yet a fourth subspecies and a full species within the taxon *T. dimidiata* [20].

In summary, phenotypic and genetic studies have indicated that *T. dimidiata* is a complex of sibling or near-sibling taxa, although the precise number of species and subspecies and their relationship with each other remain uncertain. To further our knowledge on the taxonomy and population subdivision of this important Chagas disease vector, we present new data based on two mitochondrial gene fragments, cytochrome *b* (cyt b) and nicotinamide adenine dinucleotide dehydrogenase 4 (ND4).

## Materials and Methods

### Insect Sampling and DNA Isolation

**Figure 1:**
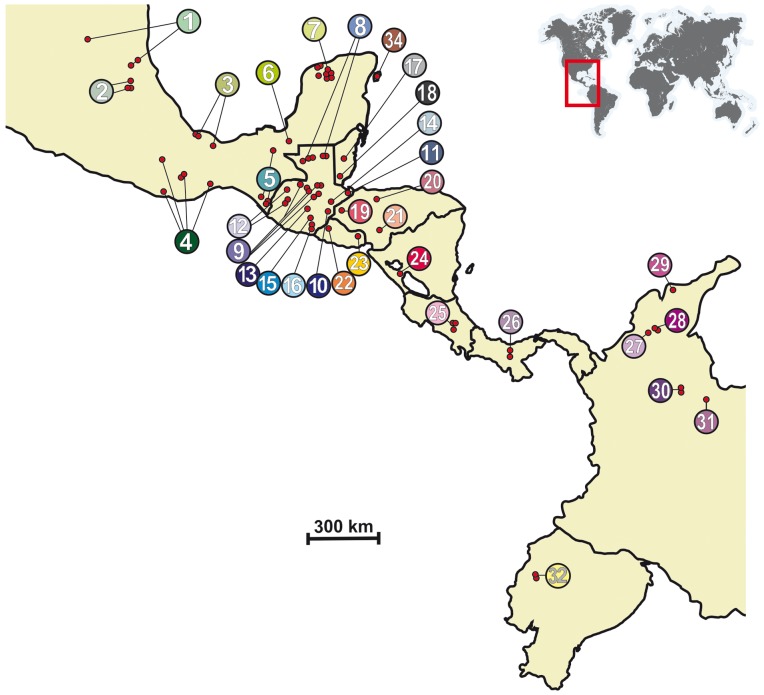
Sampling sites of representatives of the *Triatoma dimidiata* species complex. Numbers in the circles correspond to numbers of sampled locations listed in Table 1.

A total of 126 *T. dimidiata* specimens were collected from 32 localities across the species geographic range (Table 1 and Figure 1). Ten additional specimens of five closely related *Triatoma* species (*T. hegneri*, *T. flavida*, *T. phyllosoma*, *T. pallidipennis*, and *T. nitida*) were also sampled (Table 1). Insects were collected between 1995 and 2004 from domestic, peridomestic, and sylvatic habitats and identified following the Lent and Wygodzinsky [6] taxonomic key. Whenever necessary home/property owners gave consent for traps to be placed. One leg of each individual was stored in 95% ethanol until the DNA purification step. Extractions were performed using the Wizard Genomic DNA extraction kit (Promega, Madison, Wisconsin) following the manufacturer recommendations.

**Table 1:** *Triatoma* samples used in the study.

No.	Country	Map No.	Capture Location			Ecotope^1^	Sequencecodes	Cytb GenBank No.	ND4 GenBank No.
***T. dimidiata***									
**1**	**Mexico**	**1**	San Luis Potosí	Axtla de Terrazas, Temalacaco		D	MxSa1	JN585833	AF454685
**2**				San Antonio, Xolol		D	MxSa2	JN585834	
**3**						D	MxSa3	AY062149	
**4**		**2**	Hidalgo	Acomul		D	MxHi3	AY062151	AF454686
**5**				Canali		D	MxHi4	AY062151	JN620155
**6**						D	MxHi5	AY062151	JN620155
**7**		**3**	Veracruz	Mesa de Tlanchinol		D	MxVe1	AY062150	AF454685
**8**						D	MxVe2	AY062149	AF454684
**9**						D	MxVe3	AY062149	
**10**				La Luz		D	MxVe4	JN585835	JN620155
**11**		**4**	Oaxaca	Cañada Atotonilco, Los S R Nopala		P	MxOx1	JN585836	
**12**						P	MxOx2	JN585836	
**13**				San Juan Comaltepec		P	MxOx3	JN585837	JN620156
**14**				San Juan Juquila		P	MxOx4	AY062149	
**15**						P	MxOx5	JN585838	
**16**						ND	MxOx6	JN585837	
**17**						P	MxOx7	JN585836	
**18**		**5**	Chiapas	Palenque		S	MxCh1	JN585839	JN620157
**19**				Tapachula, Manacal		P	MxCh2	JN585840	JN620158
**20**						P	MxCh3	JN585841	JN620159
**21**						P	MxCh4	JN585842	JN620159
**22**				Tuxtla Chico, Medio Monte		P	MxCh5	JN585840	JN620158
**23**		**6**	Tabasco	El Rosario		D	MxTa	JN585843	
**24**		**7**	Yucatán	Yaxkukul, Rancho San Antonio		S	MxYu1	AY062162	AF454697
**25**						S	MxYu2	AY062160	AF454695
**26**						S	MxYu3	AY062163	AF454698
**27**						S	MxYu4	JN585844	
**28**						S	MxYu5	AY062158	AF454693
**29**						S	MxYu6	AY062159	AF454694
**30**						S	MxYu7	AY062161	AF454696
**31**						S	MxYu8	AY062164	AF454699
**32**			Yucatán	Carretera Paraíso-Progreso		S	MxYu9	AY062158	
**33**						S	MxYu10	JN585845	JN620160
**34**				Merida		D	F58MxY	FJ197158*	
**35**						D	F59MxY	FJ197159*	
**36**	**Guatemala**	**8**	Petén	Yaxhá		S	GuPe1	JN585846	
**37**						S	GuPe2	JN585847	
**38**						S (chultun)	GuPe3	JN585848	
**39**						S	GuPe4	JN585839	
**40**						S	GuPe5	JN585849	
**41**						S (palm)	GuPe6	JN585850	
**42**						S (palm)	GuPe7	JN585851	
**43**						S	GuPe8	JN585852	
**44**						S	GuPe9	JN585853	
**45**						S (chultun)	GuPe10	JN585854	
**46**						S (palm)	GuPe11	JN585855	
**47**						S (palm)	GuPe12	JN585856	JN620161
**48**						S	GuPe13	JN585857	JN620162
**49**						S	GuPe14	JN585858	
**50**		**9**	Alta Verapaz	Cahabón		D	GuVe1	JN585859	
**51**						D	GuVe2	JN585860	
**52**				Lanquin		S (cave)	GuVe3	JN585861	JN620163
**53**						S (cave)	GuVe4	JN585859	
**54**						S (cave)	GuVe5	JN585859	
**55**						S (cave)	GuVe6	JN585859	JN620164
**56**				Tamahú		D	GuVe7	JN585862	
**57**				Tucurú		D	GuVe8	JN585863	
**58**						ND	GuVe9	JN585864	JN620165
**59**			.			ND	GuVe10	JN585865	JN620166
**60**				Tamahú		ND	GuVe11	JN585866	JN620165
**61**				San Marcos Lachuá		P	Gua1	JN585867	
**62**						P	Gua2	JN585868	
**63**						P	Gua3	JN585869	JN620167
**64**						P	Gua4	JN585870	JN620168
**65**		**10**	Chiquimula	Tuticopote		ND	Gua7	JN585871	
**66**						ND	Gua8	JN585871	
**67**				San Juan Ermita, Chanco		D	GuCh	JN585872	
**68**		**11**	Izabal	Los Amates, Hacienda el Manacal		S (palm)	GuIz1	JN585873	
**69**						S (palm)	GuIz2	JN585874	
**70**		**12**	Quiché	Olopa		ND	Gua5	JN585871	
**71**				Tituque		ND	Gua6	JN585871	
**72**				Canillá		D	GuQu1	JN585875	
**73**				San Andrés Sajcabajá, Xepalzac		D	GuQu2	JN585876	
**74**						P	GuQu3	JN585877	
**75**		**13**	Alta Verapaz	San Marcos Lachuá		ND	GUA1B	JN585878	JN620169
**76**						ND	GUA3B	AY062157	AF454692
**77**		**14**	Zacapa	Río Hondo, El Cajón		P	GuZa	JN585875	
**78**		**15**	Santa Rosa	Amberes		D	GuSa1	JN585879	
**79**			S.Rosa de Lima	Laguna de Pereira		D	GuSa2	JN585880	
**80**		**16**	Jutiapa	Conguaco, Laguna Seca		ND	GuJu1	JN585881	
**81**				Quesada, El Tule		D	GuJu2	JN585881	
**82**				San José Acatempa		D	GuJu3	JN585874	
**83**						D	GuJu4	JN585881	
**84**						D	GuJu5	JN585881	
**85**				San José Acatempa, Tunillas		P	GuJu6	JN585882	
**86**						P	GuJu7	JN585881	
**87**	**Belize**	**17**	Cayo	Río Frio		S (cave)	Bz1	JN585883	JN620170
**88**						S (cave)	Bz2	JN585884	
**89**				Calla Creek		D	56BzCa	FJ197156*	
**90**		**18**	Toledo	San Pedro Columbia		D	54BzTo	FJ197154*	
**91**				Santa Teresa		D	55BzTo	FJ197155*	
**92**	**Honduras**	**19**	Carrizalón			D	HoCa1	JN585885	
**93**						D	HoCa2	JN585886	JN620171
**94**						D	HoCa3	JN585887	
**95**		**20**	Yoro	Yorito, Los Planes		ND	HoYo1	AY062153	AF454688
**96**						ND	HoYo2	AY062153	AF436860
**97**		**21**	Tegucigalpa.	Colonia Nueva Suyapa		ND	HoTe1	AY062152	AF454687
**98**						ND	HoTe2	AY062153	AF436860
**99**				Barrio El Bosque		ND	HoTe3	AY062154	JN620172
**100**						ND	HoTe4	AY062156	AF454691
**101**				Colonia San Miguel		ND	HoTe5	AY062154	AF454689
**102**						ND	HoTe6	AY062155	AF454690
**103**	**El Salvador**	**22**	Santa Ana	Monte Largo		D	SaSa1	JN585888	
**104**						D	SaSa2	JN585889	
**105**						D	SaSa3	JN585890	
**106**		**23**	La Unión	El Farito		D	SaLa1	JN585891	
**107**				Amapolita		D	SaLa2	JN58589	
**108**				El Farito		D	SaLa3	JN585836	JN620173
**109**	**Nicaragua**	**24**	Masaya	Masatepe		D	Nic	JN585892	
**110**	**Costa Rica**	**25**	Heredia	Santo Tomás de Santo Domingo		P	CR1	JN585893	
**111**						P	CR2	JN585894	
**112**						P	CR3	JN585894	
**113**			San José			P	CR4	JN585895	
**114**						P	CR5	JN585895	JN620174
**115**						P	CR6	JN585894	
**116**	**Panama**	**26**	Veraguas	Santa Fe, El Macho		D	Pan1	JN585896	
**117**						D	Pan2	JN585897	
**118**						D	Pan3	JN585898	JN620175
**119**				El Pantano		S (palm)	Pan4	JN585899	
**120**						S (palm)	Pan5	JN585900	
**121**	**Colombia**	**27**	Sucre	San Onofre		S	CoSu	JN585901	
**122**		**28**	Bolívar	San Fernando		S (palm)	CoBo1	JN585902	
**123**				La Margarita		S (palm)	CoBo2	JN585903	
**124**		**29**	Magdalena, Santa Marta	Cacahualito		S (palm)	CoMa1	JN585904	
**125**				Las Tinajas		S (palm)	CoMa2	JN585905	
**126**		**30**	Santander	San Joaquin		S (rock pile)	CoSa1	JN585906	
**127**				Del Carmen		S (rock pile)	CoSa2	JN585907	
**128**		**31**	Boyacá	Boavita		D	CoBy1	JN585908	JN620176
**129**						D	CoBy2	JN585909	
**130**	**Ecuador**	**32**	Manabi	El Cade		ND	Ec3	JN585910	JN620177
**131**						P	Ec5	JN585910	
***T. hegneri***									
**132**	**Mexico**	**33**	Cozumel Island		Quintana Roo, Rancho Exekalihche	P	HgC207	JN585830	JN620178
**133**						P	HgCz85	JN585831	
**134**						P	HgCz88	JN585831	
**135**						P	Heg29	JN585832	
***T. flavida***									
**136**	**Cuba**		Pinar del Río		Península Guanacahibes	S (cave)	fl4715	JX848648	JX848653
***T. phyllosoma***									
**137**	**Mexico**		Oaxaca			P	Phy446-2	JX844671	JX848649
**138**							Phy446-7	JX844672	JX848650
***T. pallidipennis***									
**139**	**Mexico**		Morelos		Chalcatzingo	D	Palli465	JX848645	
***T. nitida***									
**140**	**Guatemala**					ND	nitN30n	JX848646	JX848651
**141**	**Guatemala**					ND	nitN27	JX848647	JX848652

1 D = domestic, P = peridomestic, S = sylvatic, ND = no data.

* indicates cyt b haplotypes from Dorn *et al.* [17].

### PCR Amplification and DNA Sequencing

A fragment of the cyt b mitochondrial gene was amplified from each specimen using primers 7432F (5′ GGACGWGGWATTTATTATGGATC 3′), and 7433R (5′ GCWCCAATTCAR GTTARTAA3′) [21]. The ND4 mitochondrial gene was also amplified and sequenced for a subset of specimens using primers ND4deg01F (5′ GGSGCYTCAACATGAGCCYT 3′), and ND4b02R (5′ TAATTCGTTGTCATGGTAATG 3′) [22]. When the DNA of the sample was of poor quality, a nested PCR was performed with the primers ND4deg (5′ TCAACATGA GCCCTTGGAAG 3′), and ND4neR (5′ TAATTCGTACTCATGGTAATG 3′) [22]. An average of 1–3 µL of purified DNA was amplified in a 50 µL reaction: 5 µL 10× buffer (Promega), 4 µL dNTPs (2.5 mM each), 2 µL MgCl_2_ (25 mM), 0.5 µL Taq DNA polymerase (Promega), and 2 µL of each primer (10 pmol/µL). Amplification conditions were: 94°C for 5 min, followed by 35 cycles of 94°C for 30 sec, 47°C for 30 sec, 72°C for 1 min, and a final elongation step at 72°C for 10 min. Purification of PCR products was performed with MultiScreen PCR purification plates (Millipore) following the manufacturer recommendations. Direct sequencing of both forward and reverse sequences was performed on an ABI 3130 (Applied Biosystems) automated sequencer.

### Sequence Variation and Phylogenetic Analyses

Standard genetic diversity indices such as both nucleotide (π) and haplotype (h) diversities, and number of variable and parsimony informative sites were estimated using DNASP 5.10 [23]. Tajima’s D [24], as implemented in ARLEQUIN 3.11 [25], were used to test for mutation-drift equilibrium deviation in the overall sample.

The strategy employed to infer the phylogenetic relationships among *T. dimidiata* populations was to first generate a tree based on all cyt b sequences available, identify the main clades present, and then select at least one representative specimen of each clade to be further sequenced for the ND4 gene fragment to be used in a cyt b+ND4 combined analysis. Other *Triatoma* species (*T. flavida*, *T. phyllosoma*, *T. pallidipennis*, and *T. nitida*) were used as outgroups in the phylogenetic analyses. Best-fitting substitution models for each dataset were determined with JMODELTEST 0.1 [26], [27] based on Akaike’s information criterion (AIC [28]), which led to the selection of the Hasegawa-Kishino-Yano (HKY) model [29] with a proportion of invariable sites (+I), and gamma-distributed rate heterogeneity among the remaining sites (+G).

Phylogenies were inferred by Maximum Likelihood (ML) using PHYML 2.4.4 [30]. Bootstrap node support values were estimated from 1000 pseudoreplicates. ML trees were also submitted as user trees to MRBAYES 3.1.2 [31] for a Bayesian analysis. Posterior probabilities of phylogenetic trees were estimated by a 10,000,000-generation Metropolis-coupled Markov chain Monte Carlo (MCMC) simulation (four chains, chain temperature = 0.2) under the HKY+I+G model of substitution, with parameters estimated from the dataset. A majority-rule (50%) consensus tree was constructed following 200,000 burn-in generations to allow likelihood values to reach stationary equilibrium. Identical conditions were used for the cyt b and the combined (cyt b+ND4) datasets.

### Population-level Inferences and Divergence Times

Mean intra- and inter-group Kimura 2-parameters genetic distances [32] were estimated in MEGA5 [33], with standard errors estimated by bootstrapping (pseudoreplicates). A median-joining network analysis [34] was performed using NETWORK 4.5.1.6 (http://www.fluxus-engineering.com). The maximum number of mutations between haplotypes within the same network for cyt b was 61. The 95% connection limit was not used because of the high levels of sequence divergence, which would cause an undesired fragmentation of the network.

Principal component analysis (PCA) was used to classify all input sequences at once into one or more groups. Variable sites from the nucleotide sequence dataset were selected and nucleotide bases were coded (A = 1, C = 2, G = 3, T = 4) and combined in an alignment matrix, where each row represents a specimen’s DNA sequence. This alignment matrix was then converted into a genetic distance matrix as implemented in GENALEX 6.4 [35].

DNASP was used to generate distribution plots of pairwise sequence differences. No attempt was made to compare the observed distributions with expected distributions, because all models available in the software for producing expected distributions are suitable only for population-level analysis.

We used cyt b to estimate divergence times as we had more data for this particular gene fragment (both for specimens and haplotypes), and because reliable evolutionary rate estimates are available for this gene [36]. The time to the most recent common ancestor (tMRCA) was estimated for all major genetic groups revealed in the phylogenetic analyses using a Bayesian approach with BEAST 1.6.1 [37]. The analysis was performed using an HKY+I+G model of nucleotide substitution with gamma-distributed rate variation among sites and four rate categories – the substitution model selected using JMODELTEST. We used the suggested divergence rate of 1.1 to 1.8% per Myr [36]. The Yule process was chosen as speciation process for our data set. Results from two independent runs (20,000,000 generations, with the first 2,000,000 discarded as burn-in and parameter values sampled every 1000 generations) were analyzed with TRACER 1.5 [38] to assess convergence and confirm that the combined effective sample sizes for all parameters were >200, ensuring that the MCMC had ran long enough to produce valid estimates for the parameters [39]. The dating process was based on all specimens per group to calculate the distance (time) to the nearest node that determines each group.

## Results

### Sequence Variation and Phylogenetic Analyses

A total of 126 cyt b (621 bp long) and 47 ND4 sequences (554 bp long) were produced for *T. dimidiata*. In addition, four 4 cyt b and one ND4 sequences were generated for *T. hegneri*, along with nine sequences (three cyt b and six ND4) for the outgroup species (Table 1). Five *T. dimidiata* cyt b sequences previously reported [17] were retrieved from GenBank and also included in the analyses. There was no indication of the presence of pseudogenes or *numts* among the sequences generated as no *indels* or stop codons were detected and all sequences appeared to be evolving (i.e. accumulating mutations) as expected for normal mtDNA protein coding genes. Inspection of the *T. dimidiata* mtDNA sequences revealed the existence of 97 and 36 unique haplotypes for the cyt b and ND4 gene fragments, respectively. Basic statistics are presented in Table 2. Neutrality tests failed to reject the null hypothesis that mtDNA sequences were evolving in a neutral manner in the studied species (Tajima’s D: P>0.70; Table 2).

**Table 2:** DNA polymorphism and neutrality tests.

	cytb	ND4	Cytb+ND4
**Length (bp)**	621	572	1175
**N_seq_**	131	47	47
**N_hap_**	97	36	43
**S**	223	95	262
**P**	188	93	228
**h**	0.9938	0.9885	0.9950
**π**	0.0770	0.0554	0.0697
**Tajima’s D**	0.5544^a^	0.7549^a^	0.9460^a^

Nseq: Number of sequences; Nhap: Number of haplotypes; S: Variable sites; P: Parsimony informative sites; h: Haplotype diversity; π: Nucleotide diversity; Tajima’s D (Tajima 1989);

a P>0.70.

Saturation plots for both cyt b and ND4 gene fragments (third codon position transversion and transition substitutions against HKY+G+I distances), show no indication of saturation (results not shown).

**Figure 2:**
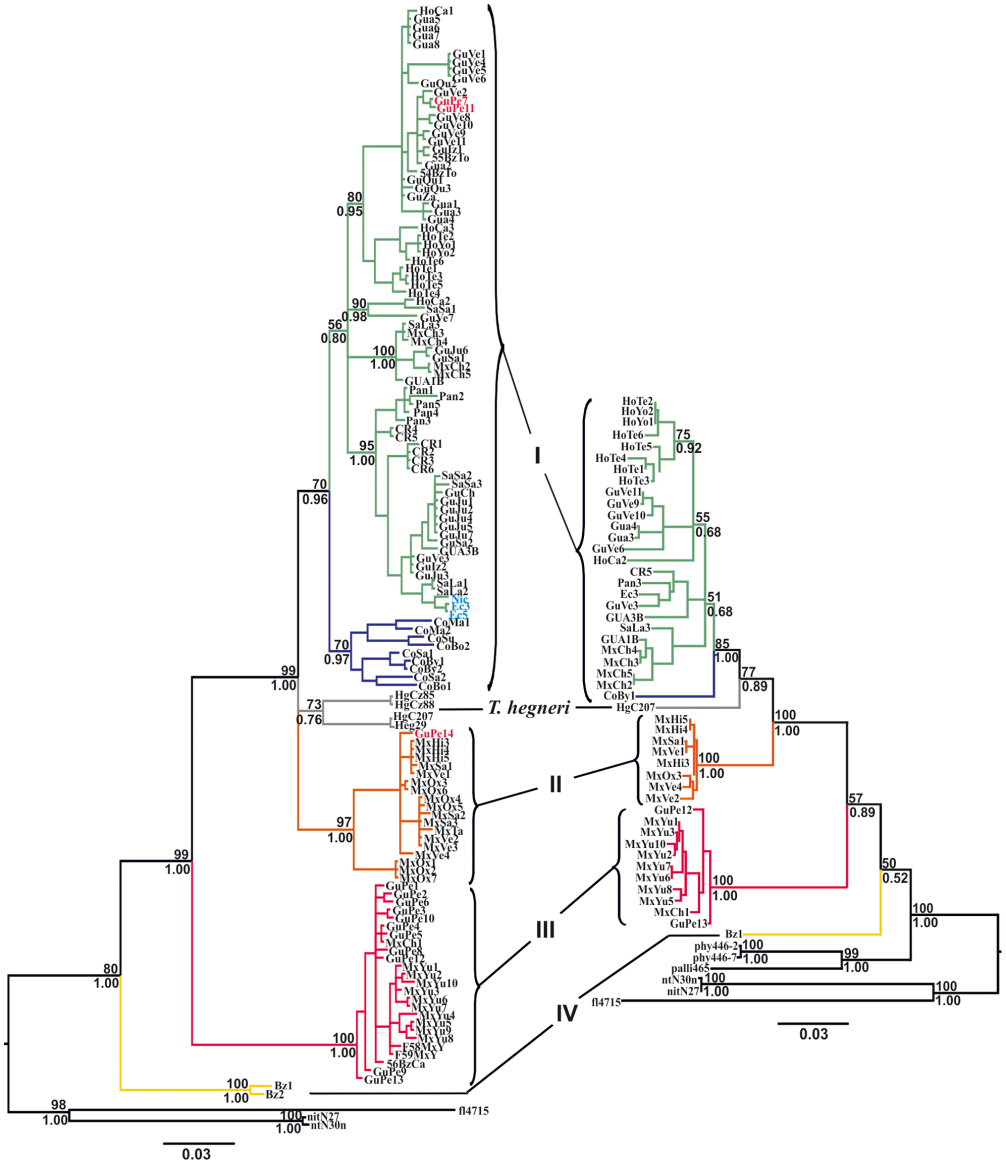
ML phylogenetic trees for *Triatoma dimidiata* species complex based on cyt b (left) and cyt b+ND4 (right) gene fragments. Bootstrap support values (above 50) are given above branches; Posterior probabilities for the Bayesian analysis are given below branches. Branch color codes indicate each of the four different genetic groups (plus *T. hegneri*) that comprise the *T. dimidiata* species complex. The three haplotypes in blue (Nic, Ec3, and Ec5) call attention to the high genetic similarity between specimens from Manabi, in Ecuador, and those from Nicaragua, indicating that *T. dimidiata* populations from the latter most likely represent the source of insects that were recently introduced into Ecuador.

ML and Bayesian phylogenetic methods yielded the same tree topologies for both datasets used (cyt b and cyt b+ND4). However, the analysis of the larger cyt b+ND4 combined dataset (1175 bp) led to the resolution of the Group I/Group II/*T. hegneri* polyphyly that was not discriminated in the cyt b tree (Figure 2a).

**Figure 3:**
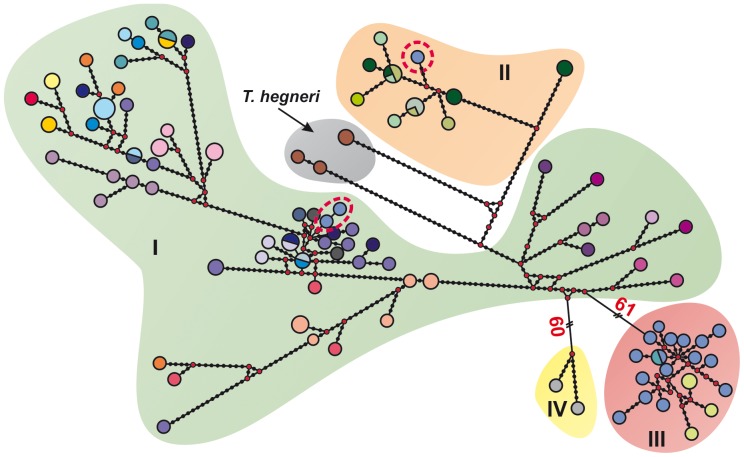
Median-joining haplotype network based on *T.* *dimidiata* and *T. hegneri* cytochrome *b* haplotypes. The size of the circles on the network is proportional to haplotype frequency. Small red circles represent missing haplotypes. Network circle colors are the same as in Fig. 1. Circles with dotted red outlines denote the presence of haplotypes from Guatemala, Petén among groups I and II.

Both cyt b and cyt b+ND4 tree topologies indicate the existence of four well-defined monophyletic groups: Group I includes samples from Mexico (Chiapas), Guatemala, Honduras, El Salvador, Nicaragua, Costa Rica, Panama, Ecuador, and Colombia; Group II comprises the westernmost samples from Mexico but also includes specimens from Tabasco and Petén; Group III includes specimens from Petén (Guatemala), Yucatán (Mexico), and Belize; and Group IV includes sylvatic samples from Belize. *T. hegneri*, from the island of Cozumel, Mexico, appears as yet another independent lineage within the range of between-group variability observed (Figures 2 and 3). Mean Kimura 2-parameters pairwise cyt b genetic distances among Groups I–IV plus *T. hegneri* were very high, ranging from 0.080 to 0.155 (Table 3).

**Table 3:** Mean K2P pairwise genetic distances between the four genetic groups revealed in the study (and *Triatoma hegneri*), for the cytochrome *b* gene fragment.

	Group I	Group II	Group III	Group IV	*Triatoma hegneri*
**Group I**	**0.045**				
**Group II**	0.088	**0.021**			
**Group III**	0.147	0.148	**0.024**		
**Group IV**	0.149	0.144	0.153	**0.018**	
***Triatoma hegneri***	0.080	0.089	0.136	0.155	**0.043**

Intergroup distances are in the lower left section; mean intragroup distances are in bold.

**Figure 4:**
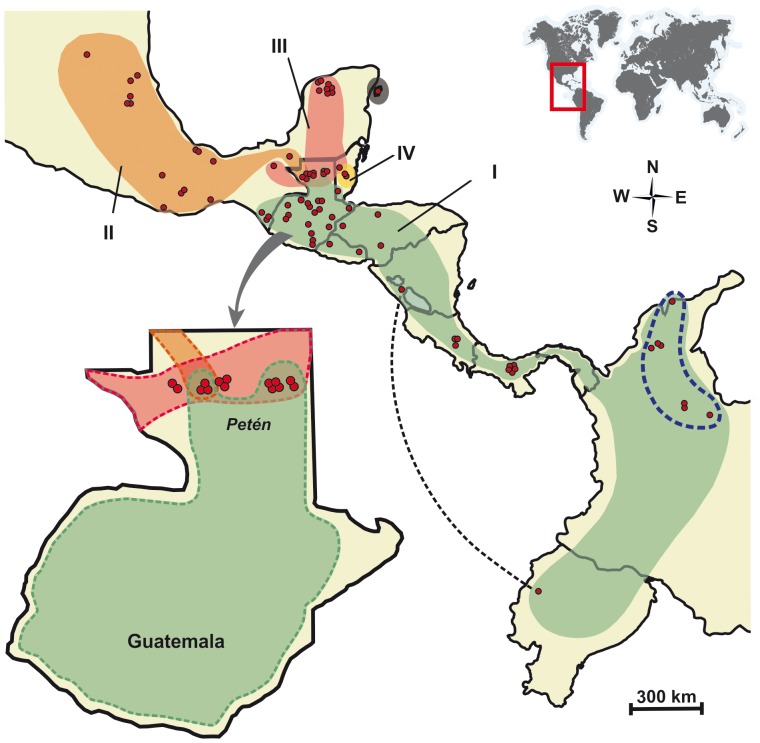
Map showing the geographic distribution of the four genetically divergent monophyletic groups identified within *Triatoma dimidiata* (plus *T.* *hegneri*) based upon Bayesian and ML phylogenetic analyses of cyt b and ND4 sequences. Group I occurs from Southern Mexico through Central America and into Colombia and Ecuador; Group II comprises samples from Western and Southwestern Mexico; Group III includes specimens from the Yucatán peninsula (excluding Belize and *T. hegneri*), Group IV includes sylvatic samples from Belize; and *T. hegneri*, from the island of Cozumel. The doted blue outline around the Easternmost sites of Group I demarcates the geographic distribution of the *T. dimidiata* samples from Colombia. The doted black line serves to indicate, based on haplotype similarity, Nicaragua as the most likely source of insects to have colonized Ecuador sometime in the recent past.

Group I is the most widely distributed and genetically variable clade (Figure 4), with within-group divergence reaching values as high as 8.5% (when Colombian haplotypes are compared with Central American haplotypes). Thus, we can suppose that it might harbor more than one species. Observation of the cyt b tree (Figure 2a) towards the lower part of Group I reveals a striking similarity between haplotypes obtained from a specimen collected in Masaya, Nicaragua (Nic) with those obtained from specimens from Manabí, in Ecuador (Ec3 and Ec5), suggesting a very recent common origin.

Specimens collected from the Lanquín caves, in Alta Verapaz, Guatemala (GuVe3, GuVe4, GuVe5, and GuVe6), are genetically similar to other *T. dimidiata* Group I insects (Figure 2a).

### Population-level Inferences and Divergence Times

With the observation of the magnitude of the inter-population divergence levels of the mtDNA sequences generated, and after unsuccessful attempts to extract meaningful population-level information from the data, we realized that it would be worthless (uninformative) to proceed with regular population-level inferences such as F_ST_ or AMOVA, and therefore decided to exclude such analyses from this paper.

The median-joining haplotype network shows that most specimens presented unique haplotypes (Figure 3). Moreover, highly divergent haplotypes were found in Petén, Guatemala, which segregated into different parts of the network. Conversely, certain haplotypes found in geographically distant (Ecuador and Nicaragua) were strikingly similar. Overall, the network displayed the same groups detected in the phylogenetic analyses. The median-joining haplotype network illustrates the intricate genetic structure that characterizes Group I.

**Figure 5:**
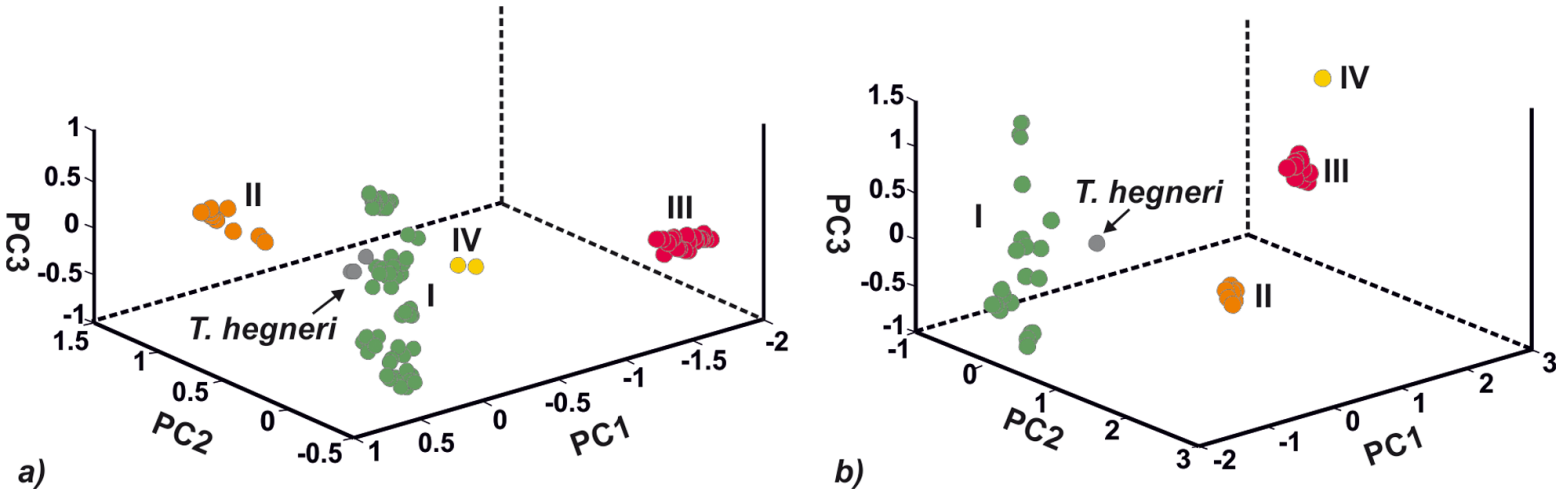
Principal component plots constructed from genetic similarity matrix based on cyt b sequences (*a*) and on cyt b and ND4 (*b*) combined data. Color codes for different groups of the *Triatoma dimidiata* complex coincide with colors used in Fig. 2.

PCA based on cyt b sequences alone revealed only three groups, with *T. hegneri* falling within Group I, while PCA of the combined cyt b+ND4 dataset resolved the same four groups (plus *T. hegneri*) recovered in the phylogenetic analyses (Figures 5a, 5b).

**Figure 6:**
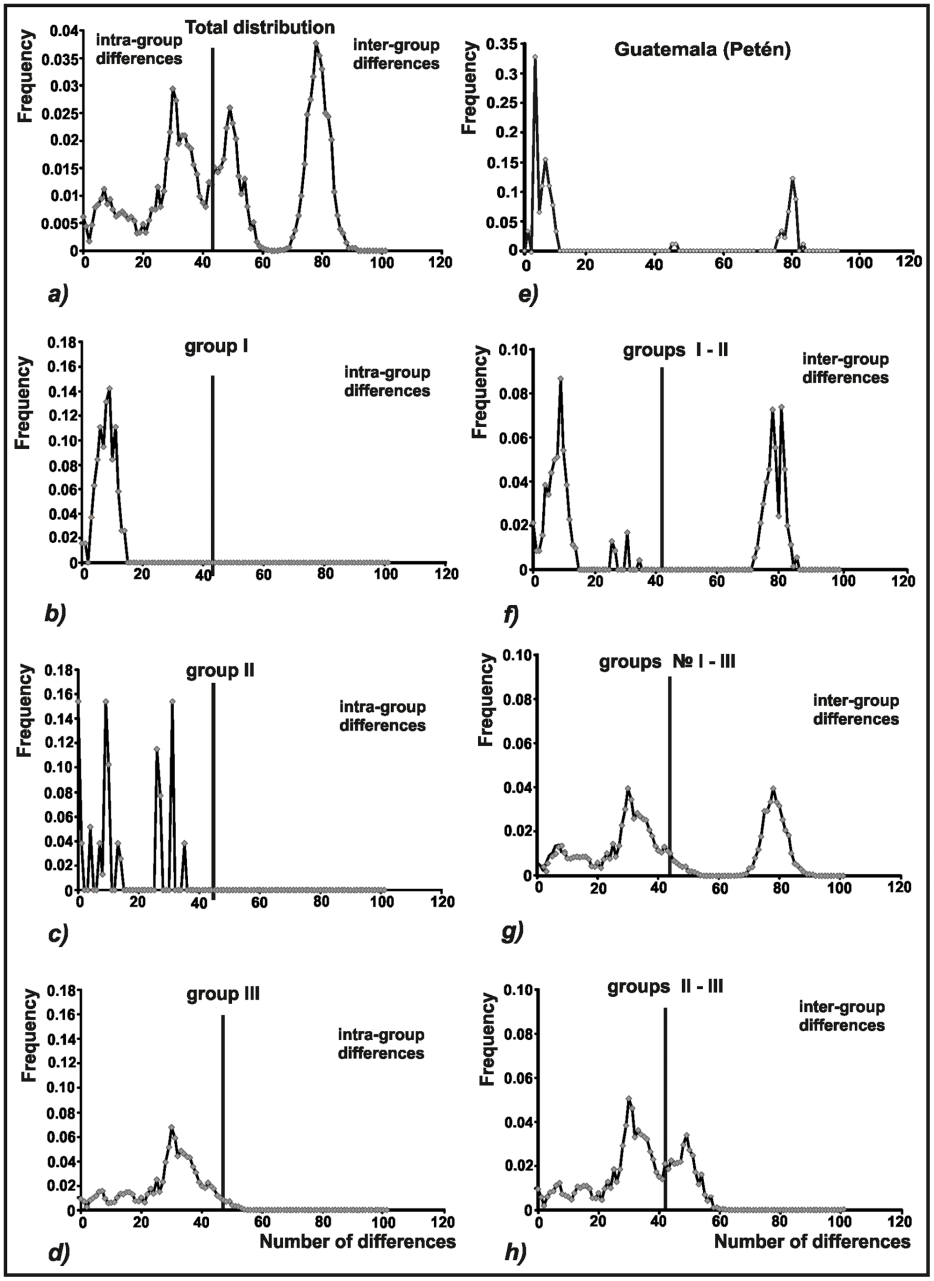
Mismatch distribution of *Triatoma dimidiata* species complex based on cyt b nucleotide sequences. (*a*) Total distribution. (*b*, *c*, *d*) Mismatch distribution within each group. (*f*, *g*, *h*) Mismatch distribution between different pairs of groups. (*e*) Mismatch distribution of haplotypes from Petén, Guatemala.

Results of within- and between-group comparative analysis of mismatch distributions are depicted in Figure 6. Mismatch distribution within Group III exhibits a unimodal distribution, with most pairwise comparisons revealing small genetic distances. Mismatch distributions within Groups I and II are multimodal and ragged, and contain a larger proportion of comparisons resulting in larger genetic distances. All inter-group mismatch distributions are clearly multimodal and have similar shapes, with most pairwise comparisons revealing large genetic distances; this same pattern is evident when including all *T. dimidiata* species complex members (Figure 6a).

Figure 6e represents the mismatch distribution for individuals from Petén, Guatemala, where representatives of Groups I, II, and III occur in sympatry. The mismatch frequency distribution is multimodal, with visibly separated peaks that reflect the absence of haplotypes with intermediate genetic distances. This clearly suggests the existence of reproductive barriers isolating these groups from one another. These Petén specimens are therefore very likely to belong to different biological species; divergence time estimates suggest that they have been evolving independently for at least about five million years (Table 4).

**Table 4:** Time of divergence within and among putative species of the *Triatoma dimidiata* complex (and 95% confidence intervals) calculated using the program Beast.

Taxa	tMRCA (95%CI)
**Group I**	2.81 (1.97–3.76)
**Group II**	1.91 (1.18–2.80)
**Group III**	0.87 (0.51–1.24)
**Group IV**	0.69 (0.27–1.13)
***Triatoma hegneri***	2.64 (1.60–3.89)
**Group I+** ***Triatoma hegneri***	3.92 (2.72–5.34)
**Group I+** ***Triatoma hegneri*** **+Group II**	4.21 (2.93–5.66)
**Group I+** ***Triatoma hegneri*** **+Group II+Group III**	7.10 (4.86–9.64)
***Triatoma dimidiata*** ** species complex**	8.25 (5.75–11.22)

tMRCA, time to the most recent common ancestor in million years before the present.

## Discussion

Since its description by Pierre André Latreille, in 1811 (as *Reduvius dimidiatus*), the taxonomy of *T. dimidiata* has been a topic of controversy (reviewed in [14]). Central to the debate was the issue of whether morphologically recognized subspecies should merit specific status. Lent and Wygodzinsky [6] put an end to the dispute by concluding, after the examination of 160 specimens from the species’ distribution range, that the differences observed were “clinal in nature”, and thus, all morphological types should be considered variations within the same species. Our results unmistakably reject this hypothesis.

We report the existence of very high levels of mitochondrial DNA (cyt b and ND4) sequence divergence among populations of *T. dimidiata* sampled throughout its geographic range. Bayesian and ML phylogenetic analyses indicate the existence of five well defined monophyletic groups, including the formally described species *T. hegneri* from the island of Cozumel. Group I stretches from Southern Mexico (Chiapas), all the way through Central America into Colombia, with Ecuadorian specimens resembling Nicaraguan material; Group II comprises samples from western and northwestern Mexico, as well as from Petén in Guatemala; Group III includes specimens from the Yucatán peninsula (including Petén, Cozumel and domestic specimens from Belize); and Group IV includes sylvatic samples from Belize (Figures 2 and 4). We will argue that each of these groups merits specific status.

### Hypothesis-testing and Taxonomic Implications

**Figure 7:**
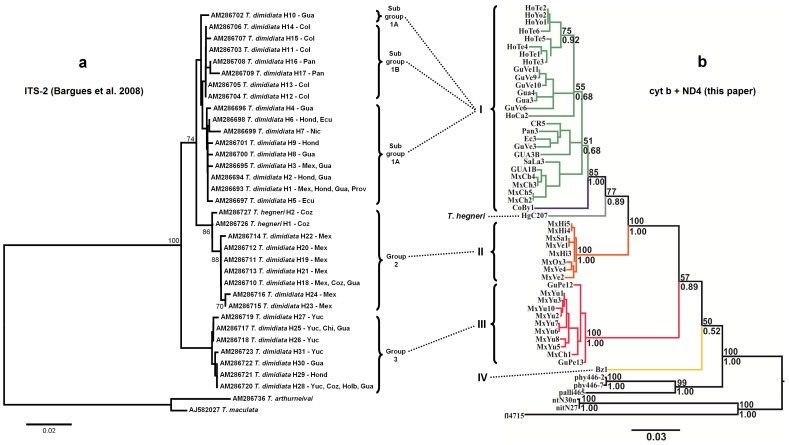
This figure shows how the topology recovered for the *T.* *dimidiata* species complex based on the phylogenetic analysis of ITS-2 sequence data of Bargues et al. [18] (a) compares to the one derived from the mtDNA sequence data (cytb+ND4) presented in this paper (b). Examination of this new figure shows that ITS-2 groups 1, 2, and 3 of Bargues et al. are essentially the same as our mtDNA groups I, II, and III (i.e. they include specimens collected from the same geographic areas). Branch color codes in “b” indicate each of the four different genetic groups (plus *T. hegneri*) that comprise the *T. dimidiata* species complex. See Discussion section for more details on the few incongruities between the two topologies and on how these were interpreted and discussed.

The comprehensive study of Bargues et al. [18] on the phylogeography of *T. dimidiata* based on ITS-2 sequences greatly advanced our knowledge on the taxonomy and evolution of this important vector. Our mtDNA-based results corroborate those of Bargues et al. [18] in the sense that they identify, overall, the same genetic groups present within *T*. *dimidiata s*.*l*. (Figure 7). However, it appears that the ITS-2 region may be too conserved to fully resolve the phylogenetic relationship among those different genetic groups [17]. By adding resolution to this matter the mtDNA gene fragments bring about a few discrepancies. For example, ITS-2 sequence data place samples from Panama together with those from Colombia, in sub-group 1B, and position *T. hegneri* specimens within group 2 (*sensu* Bargues et al. 2008 [18]). The mtDNA markers used here seem to be more appropriate for this level of taxonomic investigation. Being less conserved, and thus more informative, they allow for the detection of readily recognizable, well supported monophyletic groups.

The level of sequence divergence between groups I to IV exceeds the value of 8% reported to separate several closely related *Triatoma* species [40]. The smallest distances observed here resulted from the comparison of Groups I and II (0.088); all other inter-group comparisons gave values that surpass 13%. *T. hegneri* and Group I cyt b sequences differ by an average of 8% (Table 3).

Three distinct chromatic forms of *T. brasiliensis* from northeast Brazil analyzed with the same marker (cyt b) showed large genetic distances (*d*>0.075), which led to their recognition as members of a species complex [40]. Two of these forms were subsequently formally raised to the specific level [41], [42]. Divergence levels of the same magnitude (*d*>9%), again coupled with chromatic differences, led to the proposition that *T. rubida cochimiensis* should be considered a full species [34]. Other recent comparisons between valid *Triatoma* species based on the same marker are *T. nitida vs. T. rubida sonoriana/uhleri* (*d* = 10–11%) and *T. longipennis vs. T. recurva* (*d = *11%; [34]). In addition to the very high mtDNA genetic distances among the *T*. *dimidiata* groups here described, high values of ITS-2 sequence divergence were also reported for haplotypes belonging to groups I and II (5.62%), which, according to the authors of the study, is suggestive of speciation [18]. These are very convincing arguments in favor of the hypothesis that *T. dimidiata* is a true species complex.

Group I is the most geographically widespread and genetically variable. Pairwise within-group genetic distances can be as high as 8.5%. The divergent samples from Colombia appear as a monophyletic sister clade with respect to all other specimens in the group (which are predominantly from Central America). Colombian specimens were once described as *T. dimidiata capitata* on morphological grounds [43], to be later synonymized [6]. Thus, it is fair to speculate that this group might conceal yet another biological species.

### Sympatric Occurrence of Different Genetic Groups

Sympatry between Groups II and III is well documented in the Yucatán peninsula [17]. Although there seems to be extensive hybridization, reproductive isolating barriers (RIBs) do exist (such as reduced viability of female hybrids [44]) and appear to prevent the two species from merging into a single entity. This is a compelling argument in favor of the validation of Group III insects as a different species, as previously suggested [16], [18].

Remarkably, in Petén, Guatemala, there is not only overlapping occurrence of Groups II and III as in Yucatán, but also of Group I insects (Figure 4). Mismatch distribution results reveal multimodality caused by the absence of haplotypes with intermediate genetic distances among groups (Figure 6). This is a very significant finding as it points to the probable existence of RIBs for all combinations among these three groups, lending further support to their recognition as different biological species.

### Divergence Times and Biogeography

The Isthmus of Tehuantepec is known to represent an important recent geological barrier for a number of sister taxa of birds, mammals, and butterflies [45]. It has been shown to be a phylogeographical barrier to both highland [46] and lowland species [47]. Given the present distribution of the genetic groups revealed by the mtDNA fragments analyzed in this study, it is tempting to speculate that the Isthmus of Tehuantepec orogeny split the original population and caused the allopatric generation of Groups I and II.

The isolation that might have led to the origin of Group III insects from the Yucatán peninsula could be explained by changes in climate and vegetation that took place particularly during the Pleistocene period. Lee [48] suggests that a period of Pleistocene aridity, during which there was a continuous subhumid to xeric habitat, extended from the Pacific side of Mexico across the Isthmus of Tehuantepec to the gulf coast and from there to the Yucatán Peninsula. The increase in humidity together with the introduction of mesophytic vegetation in the area resulted in an isolation of this subhumid environment from the west of Mexico, leading to speciation.

### Triatoma Dimidiata in Ecuador

Bargues et al. [18] proposed that Ecuadorian *T. dimidiata* populations may have derived from recently introduced specimens originally from the Guatemala-Honduras-Nicaragua region, as a result of human migrations. This view was further supported by subsequent molecular analyses [15], [18] and by ecological and biogeographic observations, including the absence of records of wild populations in Ecuador (in contrast with abundant observations elsewhere) and the discontinuous distribution of the species, with Ecuadorian populations isolated from their Colombian relatives by the Central Colombian Massif and the humid Chocó eco-region [10]. The fact that *T. dimidiata* populations seem to have disappeared from some formerly infested rural areas of Ecuador [49], [50] and appear to persist only in a few urban foci (Abad-Franch F, pers. obs.) also matches the predictions of the artificial introduction hypothesis. As shown in the cyt b tree (highlighted in blue on Figure 2a) and the haplotype network (Figure 3), there is a striking similarity between haplotypes obtained from a specimen collected in Masaya, Nicaragua (Nic) and from Ecuadorian material (Ec3 and Ec5). This genetically pinpoints *T. dimidiata* populations from Nicaragua as the most likely source of insects to have colonized Ecuador sometime in the recent past.

### Lanquín Cave Specimens

Studies based on morphometry [51], RAPD [52], antennal sensilla [53] and cuticular hydrocarbons [20], [54] of cave-dwelling specimens from Lanquín, Alta Verapaz, in Guatemala, revealed great phenotypic divergence from all other *T. dimidiata* populations analyzed. The differentiation was so remarkable that it was suggested that these insects could represent an incipient species [51], [54]. A different interpretation was put forth by Bargues et al. [18], based on the phylogenetic analysis of the ITS-2 region of the rDNA, that these specimens would have derived from the ancestor which gave rise to the subspecies *T. d. dimidiata*. Yet another result, also derived from the ITS-2 marker, contradicts the former and depicts Lanquín samples as a separate independent lineage [17]. Our results indicate that the Lanquín cave specimens are no different from other *T. dimidiata* Group I specimens from Central America (see haplotypes GuVe4, GuVe5, and GuVe6 in the upper portion of Group I, and GuVe3 close to haplotypes GuIz2 and GuJu3 in Figure 2). This suggests that Lanquín cave-dwelling specimens represent a striking case of phenotypic plasticity, most likely related to micro-habitat adaptation, within a single genetic cluster.

### Samples from Belize

Sylvatic specimens from Belize (Cayo District) represent a different species which constitutes the most basal lineage of the *T. dimidiata* species complex, as previously suggested based on cuticular hydrocarbon patterns [20]. Divergence time estimates show that this lineage has been evolving independently for approximately 8.25 My (Table 4). These insects are clearly different from the domestic Belize specimens studied by Dorn et al. [17], which belong in Groups I and III (Figure 2). A possible explanation for this incongruence is that the specimens we studied were collected in the Rio Frio Cave. Interestingly, unlike the specimens collected from the Lanquín caves in Guatemala, these insects are quite large and present lighter tegument coloration throughout all developmental stages (Marcet PL, Dotson EM, pers. obs.).

### Concluding Remarks

Bargues et al. [18] state, in the Discussion section of their paper, that – “Results of the present study do not support the rise of the abovementioned subspecific taxa to species level for the time being, although it is evident that in the three cases relatively long divergence processes have taken place. Similar genetic studies with other molecular markers may contribute to a more complete assessment of these evolutionary isolation and speciation processes.” We believe we have made an important contribution toward that end. The data presented here unmistakably reject the hypothesis that the intraspecific variation seen in *T. dimidiata* is clinal. The results further support previous analyses that indicated the existence of clearly recognizable genetic groups within *T. dimidiata*. We report the finding of very high levels of mitochondrial DNA (cyt b and ND4) sequence divergence among monophyletic populations of this vector which are incompatible with current views that regard most of these populations (with the exception of the Yucatán clade) as subspecies. We alternatively defend the interpretation that all four genetic groups revealed herein merit specific status. All the evidence presented strongly supports the proposition that *T. dimidiata* is a complex of five species (as it also includes *T. hegneri*) that play different roles as vectors of Chagas disease, from the apparently strictly sylvatic populations of Group IV in Belize to the heavily synanthropic populations (Groups I and II) in Mesoamerica, Colombia and Ecuador – and with the Yucatán clade (Group III) apparently presenting intermediate behavior.
