# Correction: Phylogeographic Pattern and Extensive Mitochondrial DNA Divergence Disclose a Species Complex within the Chagas Disease Vector *Triatoma dimidiata*

**DOI:** 10.1371/annotation/7881bf8a-21fe-40d1-9e23-3e7edee8b782

**Published:** 2013-10-01

**Authors:** Fernando A. Monteiro, Tatiana Peretolchina, Cristiano Lazoski, Kecia Harris, Ellen M. Dotson, Fernando Abad-Franch, Elsa Tamayo, Pamela M. Pennington, Carlota Monroy, Celia Cordon-Rosales, Paz Maria Salazar-Schettino, Andrés Gómez-Palacio, Mario J. Grijalva, Charles B. Beard, Paula L. Marcet

The correct affiliation for Elsa Tamayo (affiliation number 6) is:

Instituto Nacional de Salud Pública, Centro de Investigaciones sobre Enfermedades Infecciosas, Departamento de Diagnóstico Epidemiológico, Cuernavaca, Morelos, México.

